# Exercises plus intra-articular injection for knee osteoarthritis: a systematic review with meta-analysis

**DOI:** 10.1186/s42358-025-00458-3

**Published:** 2025-12-10

**Authors:** Christine Brumini, Jamil Natour, Sandra Mara Meireles Adolph, Rita Nely Vilar Furtado, Anamaria Jones

**Affiliations:** Universidade Federal de Sao Paulo/Escola Paulista de Medicina (UNIFESP/EPM) – Disciplina de Reumatologia, Rua dos Otonis, 863 - Vila Clementino, São Paulo, SP CEP 04025-002 Brazil

**Keywords:** Osteoarthritis, knee, Aged, Exercise therapy, Injections, Intra-articular, Arthralgia

## Abstract

**Objective:**

To evaluate whether the combination of exercise and intra-articular injection (IAI) effectively improves pain, function, and quality of life in patients with knee osteoarthritis (OA) compared to any control group in the short, medium, and long term through a systematic review.

**Methods:**

A comprehensive search strategy was applied in the databases Embase, PubMed, MEDLINE, CENTRAL, CINAHL, and PEDro. Inclusion criteria focused on randomized controlled trials examining the effects of exercise combined with IAI in patients with knee OA, with outcomes assessed at short-, medium-, and long-term follow-ups. The primary outcomes were pain and function. The quality of the evidence was evaluated using the GRADE system.

**Results:**

Eleven studies, comprising 802 participants, were included. All studies investigated the combination of IAI and exercise. A statistically significant difference in pain was observed: in the short and medium term, the Botulinum toxin IAI group demonstrated superior pain reduction compared to the Hyaluronic acid IAI group (MD -1.32, 95% CI -2.20 to -0.44 and MD -9.09, 95% CI -13.16 to -5.01, respectively). In the medium term, Saline IAI was more effective than Corticosteroid IAI (MD 1.99, 95% CI 0.49 to 1.90). Regarding function, Saline IAI outperformed IAI with any medication in the short term (MD 0.50, 95% CI 0.20 to 0.79). In terms of quality of life, the Corticosteroid IAI group demonstrated superior physical function and mental health outcomes compared to the Saline IAI group in the medium term (MD -0.43, 95% CI -0.77 to -0.08 and MD -0.38, 95% CI -0.76 to -0.01, respectively). In the long term, physical function improved more with IAI combined with exercise compared to exercise alone.

**Conclusion:**

Given the very low quality of the evidence, it is not possible to definitively conclude that the combination of IAI and exercise is more effective than IAI or exercise alone in patients with knee OA. Further high-quality studies are needed to establish more definitive conclusions.

**Registration:**

The International Prospective Register of Systematic Reviews (PROSPERO): CRD42021277729.

**Clinical trial number:**

Not applicable.

**Supplementary Information:**

The online version contains supplementary material available at 10.1186/s42358-025-00458-3.

## Background

Knee osteoarthritis (OA) is a chronic and progressive condition characterized by joint pain that is exacerbated by movement, morning stiffness lasting less than 30 min, joint effusion, crepitus, and restricted range of motion. These symptoms significantly impair daily activities and quality of life, particularly among older adults. Functionally, approximately 80% of individuals with knee OA experience some degree of movement limitation, with 25% being unable to perform most activities of daily living. The prevalence of knee OA continues to rise due to an aging population and increasing rates of obesity, underscoring the need for effective management strategies [1, 2].

Guidelines for knee OA management emphasize non-pharmacological interventions as foundational therapies. These include exercise, weight management, patient education, and assistive devices such as orthoses or walking aids [3]. Exercise, in particular, is strongly recommended for its benefits in pain reduction and functional improvement, with evidence supporting a range of modalities, including aerobic training, resistance exercises, and neuromuscular strategies [4, 5]. Pharmacological interventions, such as nonsteroidal anti-inflammatory drugs (NSAIDs) and intra-articular injections (IAIs), are often employed as adjunct therapies to manage symptoms and delay disease progression [6].

Despite the individual efficacy of exercise and IAIs, recent studies have explored their combined effects with varying outcomes. A 2023 scoping review examined studies comparing the combination of exercise and intra-articular knee injections with exercise alone for knee OA management [7]. The review concluded that most included studies did not find a superior effect from adding a knee injection to exercise compared to exercise alone. However, the authors noted a paucity of literature investigating multimodal approaches, indicating a need for further research in this area.

In contrast, a 2023 randomized trial assessed the longer-term effects of the Good Life with Osteoarthritis in Denmark exercise and education program relative to an open-label placebo in individuals with knee OA [8]. The study found that both interventions provided minor long-term benefits with no significant group differences, suggesting that exercise and education may offer comparable benefits to placebo treatments in certain contexts.

Furthermore, a 2022 study compared exercise therapy and patient education to intra-articular saline injections in the treatment of knee OA [5]. The findings indicated that exercise therapy and education were as effective as saline injections in managing symptoms, highlighting the potential of non-invasive interventions for knee OA treatment.

Additionally, a 2022 study explored the effects of intra-articular botulinum toxin injections combined with therapeutic exercise [9]. The study demonstrated that this combination was more effective than hyaluronic acid injections for reducing pain and improving function in individuals with knee OA.

These recent studies underscore the ongoing exploration of combining exercise with various intra-articular injections in knee OA management. While some findings suggest no additional benefit from combining treatments, others indicate potential advantages, particularly with novel approaches such as botulinum toxin injections [10, 11]. This highlights the need for further research to establish standardized guidelines and optimize treatment strategies for individuals with knee OA.

Given the strong recommendation for both exercise and IAIs as treatments for knee OA, this systematic review aims to evaluate their combined effects comprehensively. Specifically, this study seeks to determine whether the integration of exercise with IAIs provides superior outcomes compared to either intervention alone or minimal care, with analyses conducted over short-, medium-, and long-term follow-ups.

## Methods

This study followed the Cochrane Handbook [12] and was reported in alignment with PRISMA (Preferred Reporting Items for Systematic Reviews and Meta-Analyses) guidelines [13]. This study was registered with the Prospective Register of Systematic Reviews (PROSPERO) under CRD42021277729.

### Selection criteria

#### Study types

Randomized controlled trials (RCTs) comparing exercise plus intra-articular injection (IAI) to any control group, including minimal interventions (e.g., usual care) or exercise or IAI alone, and addressing at least one predefined outcome were included. No restrictions were applied regarding date, language, or study format (conference abstracts, unpublished studies, theses, etc.).

#### Patients

Adults aged 18 years or older diagnosed with knee osteoarthritis based on the American College of Rheumatology criteria¹ were included.

#### Interventions

RCTs incorporating exercise combined with IAI were included in the intervention group, while the control group received minimal intervention, usual care, exercise, or IAI alone. No restrictions were placed on the type of strengthening exercises, frequency, session count, intervention duration, or the type of drugs used in IAI. Differences among experimental interventions were analyzed in subgroup analyses as applicable.

#### Outcomes

Primary outcomes assessed were pain and function, while secondary outcomes included quality of life, range of motion, and adverse effects. Studies evaluating short-term (up to 4 weeks), medium-term (5 to 14 weeks), and long-term (over 14 weeks) treatment effects were analyzed.

### Search strategy

A highly sensitive search strategy was implemented without restrictions regarding date, language, or publication format (up to June 2023) in the following databases: Embase, PubMed, MEDLINE, CENTRAL, CINAHL, and PEDro (Appendix 1). Additional searches included ongoing studies, reference lists of included studies, and relevant conference proceedings.

### Data extraction and quality assessment

Identified studies were organized using EndNote and Rayyan. Two reviewers independently (CB and AJ) selected relevant titles and abstracts. The same reviewers independently screened full texts for inclusion, with disagreements resolved by two additional reviewers (SMMA and JN).

### Assessment of risk of bias in included studies

Using a standardized extraction form, reviewers (CB and AJ) independently recorded bibliometric data, methods, participant characteristics, interventions, outcomes, conflicts of interest, funding, records, results, and conclusions. The risk of bias was assessed using criteria from the Cochrane Handbook and the PEDro scale [12, 14, 15]. Disagreements were resolved by SMMA and JN.

### Statistical analysis

Quantitative data were analyzed using Review Manager 5.4.1. Only continuous outcomes were considered. Treatment effects for outcomes measured on the same scale were described using mean differences (MD), while those on different scales were described using standardized mean differences (SMD). Cohen’s interpretation was applied to SMD: SMD < 0.5 (small effect), SMD 0.5 to 0.8 (medium effect), and SMD > 0.8 (large effect) [12, 16].

#### Assessment of heterogeneity

Heterogeneity was assessed using the I^2^ statistic. Data were synthesized through meta-analyses when outcomes were comparable in terms of population, intervention, evaluation, and study design, and when at least 80% of the collected results were included in the final analyses [12]. Anticipating statistical heterogeneity, a random-effects model was used, and mean differences were reported for effect size when possible.

#### Assessment of subgroup, sensitivity, and publication bias

Subgroup, sensitivity, and publication bias assessments were not performed due to the limited number of studies (maximum of four) in each meta-analysis, resulting in insufficient data.

#### GRADE assessment

Two reviewers (CB and SMMA) applied the Grading of Recommendations Assessment, Development, and Evaluation (GRADE) system to rate the quality of evidence and certainty of treatment recommendations using the GRADEpro tool [12].

## Results

**Fig. 1:**
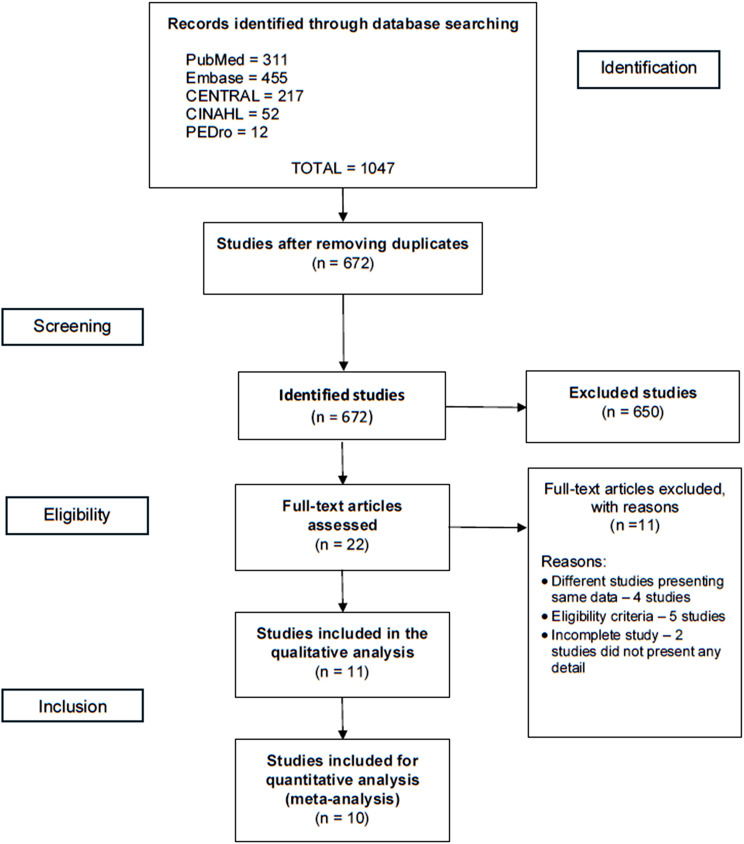
PRISMA flowchart: identification, screening, eligibility, and inclusion

The search yielded 1047 studies. Of these, 22 were selected for full-text reading, leading to the final inclusion of 11 studies for qualitative analysis and 10 studies for meta-analysis. Four studies [17–20] elicited disagreements between the authors (CB and AJ) in the inclusion decision, which were resolved through consultation with two other review authors (SMMA and JN) (Fig. 1). The reasons for excluding studies were as follows: studies with overlapping data with research already included in the review [17–20]. In addition, 6 studies did not meet the inclusion criteria, as they were not RCTs [21, 22] included additional physical modalities [23, 24] (US or shortwave diathermy) alongside exercise, and did not include exercise in combination with IAI as an intervention [25, 26].

A total of 802 patients, from studies published between 2006 and 2022, were included in this review [27, 28]. The sample size ranged from 13 to 157 patients, with 68.4% being women. In the groups where IAI was combined with exercise as an intervention, the mean age was 64.6 years, whereas in the control groups, it was 64.7 years. The included studies evaluated treatment in the short term (up to 4 weeks), medium term (5 to 14 weeks), and long term (over 14 weeks).

Regarding the outcomes, there was substantial heterogeneity between the studies. Among them, the main instruments used for evaluation were: the visual analogue scale (VAS), the Western Ontario and McMaster Universities Arthritis Index (WOMAC), and the Knee Injury and Osteoarthritis Outcome Score (KOOS) (pain domain) for assessing pain; WOMAC (function and total domains) and KOOS for functional outcomes; and the Short Form-36 Health Survey Questionnaire (SF-36) and KOOS (knee-related quality of life domain) for quality of life.

The exercise programs in the included studies were always guided by a trained professional, administered either individually or in groups, and conducted either at home [27, 29–33] or in person [11, 28, 34], lasting from 6 to 24 weeks, with a frequency of 2 to 5 times per week and session durations ranging from 30 to 45 min.

All included studies followed protocols involving quadriceps muscle strengthening exercises, which were performed using either isometric or isotonic techniques. Additionally, some studies incorporated strengthening exercises for other lower limb muscle groups [10, 11, 28–30, 34], while others included stretching and/or additional exercises such as balance and functional training [11, 29, 30, 33–35]. Three studies prescribed post-IAI ice pack use, if necessary [11, 31, 32]. Regarding exercise frequency, isometric strengthening involved 5- to 10-second contractions, performed in 2 to 3 sets of 10 repetitions per day or 3 times per week. Only one study specified a 2-second rest interval between contractions. For isotonic strengthening, participants performed 3 to 5 sets of 8 to 12 repetitions, 2 to 3 times per week.

The medications used in the IAIs included corticosteroids (CS), administered in four studies [27, 28, 32, 34]. Dosages varied as follows: 1 mL (40 mg) of methylprednisolone acetate (Depo-Medrol), 1 mL (5 mg + 2 mg) of betamethasone dipropionate + betamethasone sodium phosphate, 3 mL (30 mg) of triamcinolone “Adcortyl,” and 3 mL (60 mg) of triamcinolone hexacetonide, all diluted with lidocaine and administered as a single application. Hyaluronic acid (HA) was used in four studies [10, 11, 29, 35], with only two specifying the dosage (2 mL) and three indicating the brand. HA IAIs were administered in 3 to 5 applications on a weekly basis. Platelet-rich plasma (PRP) was utilized in three studies [30–32], with administration varying between 1 and 3 applications at intervals of 1 to 4 weeks. Botulinum toxin (Botox) was applied in two studies [11, 35], at a dosage of 100 units of botulinum neurotoxin type A (BoNT-A) dissolved in saline solution, as a single injection. Dextrose IAIs were used in two studies [33, 35], one of which involved both intra- and extra-articular injections, administered in 1 to 3 weekly sessions. Saline was used as a control in four studies [11, 18, 33, 34], with one study [17] utilizing intra- and extra-articular saline injections, administered in three weekly applications, while the others employed a single application.

Regarding OA severity, six studies [10, 27–29, 33, 34], included patients with mild to moderate OA, three studies [14–16] assessed moderate to advanced OA, and two studies [11, 30] included patients with mild to advanced OA. All studies followed the Kellgren and Lawrence (K/L) radiological grading classification. Additionally, three studies [11, 34, 35] used ultrasound to guide IAI administration.

Authors who reported adverse events indicated that both the IAI intervention and exercise were safe. Regarding the exercise program, only one study [29] noted that a patient withdrew due to an inability to tolerate the program caused by a pre-existing abdominal wall hernia. Regarding IAI, three studies [30–32] reported adverse events related to PRP, including transient increases in pain and swelling lasting from three days to two weeks. Furthermore, some patients experienced transient knee stiffness, local pelvic pain, and a bloating sensation. One patient reported mild knee erythema. Four studies [10, 11, 28, 35] reported no adverse events, while three studies [27, 33, 34] did not provide this information.

Three studies [28, 33, 34] assessed adherence to the exercise program, reporting high adherence (above 78% of the prescribed sessions). Eight studies [10, 11, 29–31, 37] did not report adherence or failed to follow up, as they involved home-based exercises. Among the included articles, only three [11, 28, 34] mentioned registration on ClinicalTrials.gov. Funding was received from development agencies in four studies [21, 27, 30, 34], whereas four studies [28, 32, 33, 35] reported receiving no funding, and three studies [10, 29, 31] did not mention funding sources.

**Fig. 2:**
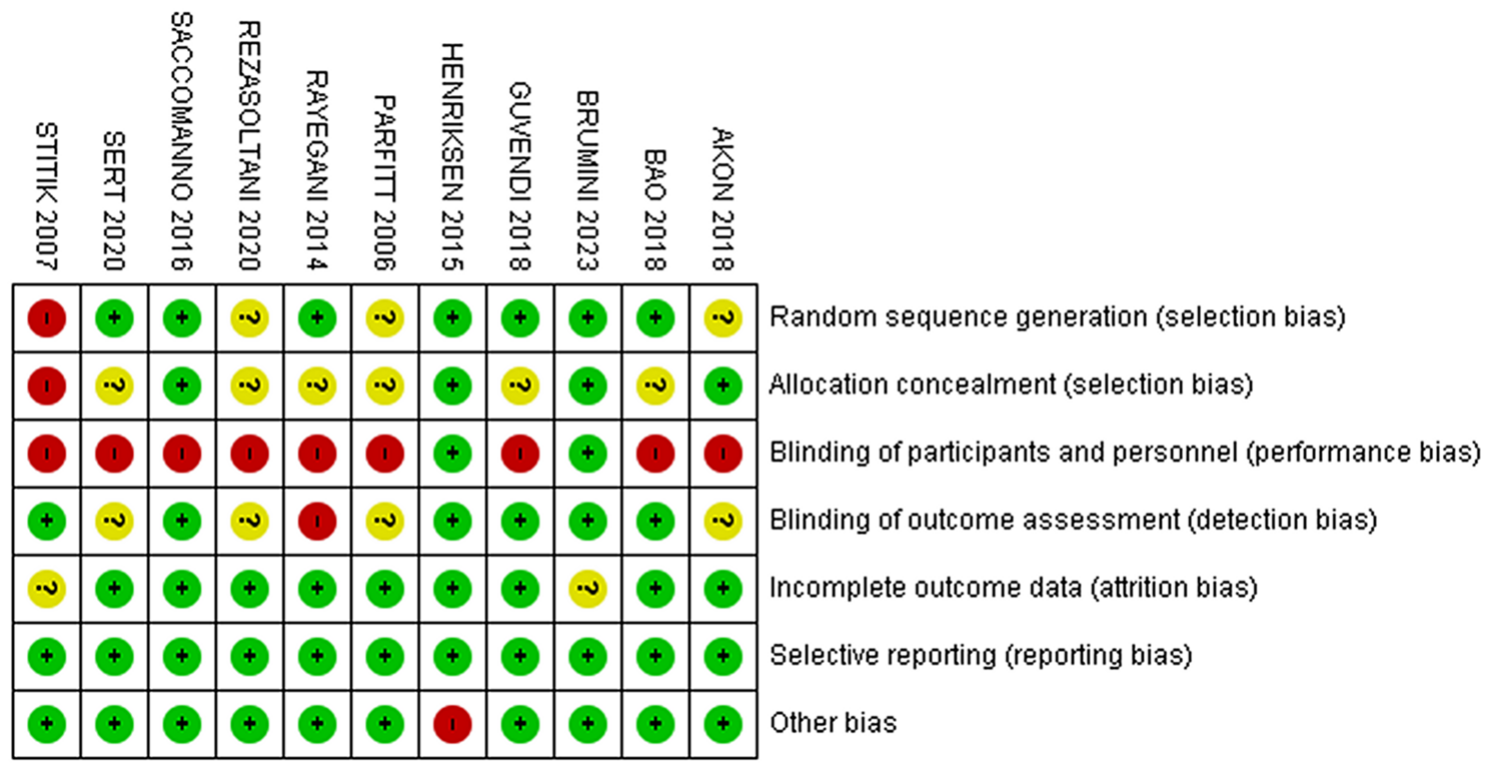
Summary of bias risk: authors’ assessment for each bias risk item for each included study

**Fig. 3:**
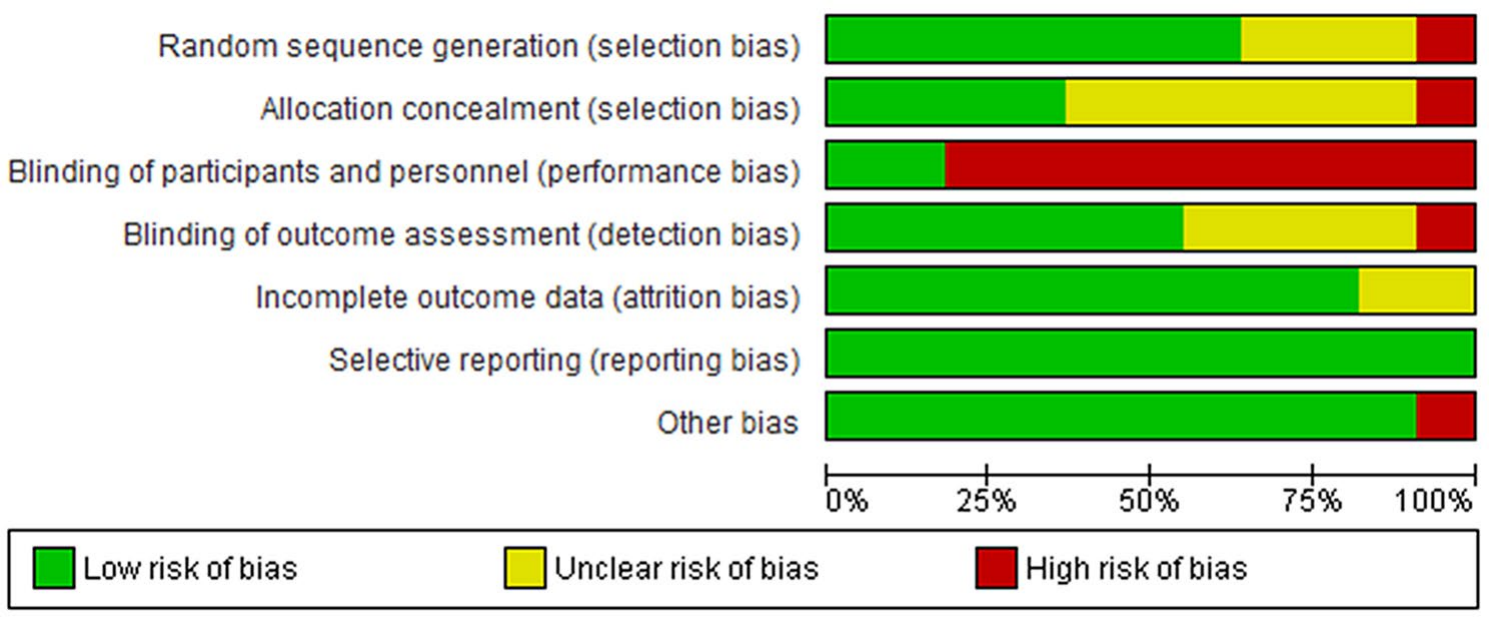
Bias risk graph: authors’ assessment of each bias risk item presented as percentages across all included studies

The qualitative details of the studies are summarized in Table 1. Figures 2 and 3 present the risk of bias assessment by the review authors using the Cochrane Collaboration tool. Table 2 provides the PEDro scale analysis and corresponding risk of bias scores. There were no disagreements among the authors regarding this analysis. Most studies exhibited a low risk of bias, with PEDro scale scores of ≥ 6 [14, 15].

**Table 1:** Qualitative analysis of included studies

Studies	Participants	Assessment measures	Interventions	Main findings
**Parfitt et al. (2006)** [27] **RCT** **Full article** **Location**: United Kingdom **Language**: English	**N**: 13 participants **EG**: • **N**: 8 • **Gender**: 7 F:1 M • **Mean age**: 76.1 years **CG**: • **N**: 5 • **Gender**: 3 F:2 M • **Mean age**: 67.2 years **Condition**: Mild to moderate OA	**Follow-up**: 2 and 8 weeks after the intervention **Assessment Measures**: • Visual Analog Scale (VAS) • WOMAC (pain and function domains) • Goniometry • Timed Up and Go Test (TUG) • 15-meter walk test	**Experimental Group**: 1 intra-articular injection (IAI) / corticosteroid (CS) + home exercise **Control Group (CG)**: 1 intra-articular injection (IAI) / corticosteroid (CS) **Home Exercise**: Guidelines for quadriceps muscle strengthening exercises with gradual loading (daily exercise chart) to be performed on the 2nd, 4th, and 6th weeks after the IAI. **IAI/CS**: 30 mg (3 ml) triamcinolone “Adcortyl” combined with 1 ml (1% lidocaine). Arthrocentesis was performed if necessary. **Duration**: 8 weeks.	Small Sample Size Analysis conducted with 12 participants (mean and standard deviation) The small number of cases in both groups did not allow for a statistically significant analysis of the obtained data. **Exercise adherence**: Not reported **Adverse events**: Not reported
**Stitik et al. (2007)** [29] **RCT** **Full article** **Location**: United States **Language**: English	**N**: 60 participants **G1**: • **N**: 20 • **Gender**: Not reported • **Mean age**: 62.5 years **G2**: • **N**: 20 • **Gender**: Not reported • **Mean age**: 64.7 years **G3**: • **N**: 20 • **Gender**: Not reported • **Mean age**: 65.5 years No statistical difference between groups regarding gender. **Condition**: OA (K/L grades 2 and 3)	**Follow-up**: Baseline, 1, 3 weeks, and 1, 3, 6, 9, and 12 months after the IAI. **Assessment Measures**: • Visual Analog Scale (VAS) (after a 15-meter walk) • WOMAC • Safety of IAI	**G1**: 3 intra-articular injections (IAI) / hyaluronic acid (HA) **G2**: 3 intra-articular injections (IAI) / hyaluronic acid (HA) + PED **G3**: 5 intra-articular injections (IAI) / hyaluronic acid (HA) **PED**: 2 instructor-guided exercises (quadriceps muscle strengthening + squats). Performed with 3 to 5 sets of 8 to 12 repetitions on alternate days. **IAI/HA**: Patients received a total of 3 or 5 intra-articular injections (IAI) weekly (every 5–9 days).	**VAS Results**: • Groups 2 and 3 showed pain improvement by week 2, while Group 1 improved by week 7. • Group 2 had both clinical and statistical improvement compared to the other groups. • Pain improvement in Group 2 continued from week 2 to week 52. Group 1 did not show improvement by week 39. **WOMAC Results**: • All groups improved (pain, function, and total) by week 52. • Group 2 demonstrated greater improvement than Group 1 in pain and function by week 52. • No statistical differences were found for the stiffness domain. **Adverse Events**: No serious adverse events were reported. One patient could not tolerate the PED due to a pre-existing abdominal wall hernia and withdrew from the study. **Exercise Adherence**: Data from exercise diaries could not be analyzed. Approximately 50% of patients did not return or complete their final diaries as instructed.
**Rayegani et al. (2014)** [30] **RCT** **Full article** **Location**: Iran **Language**: English	**N**: 62 participants **EG**: • **N**: 31 • **Gender**: 29 F:2 M • **Mean age**: 58.07 years **CG**: • **N**: 31 • **Gender**: 29 F:2 M • **Mean age**: 54.68 years **Condition**: OA (K/L grades 1 to 4)	**Follow-up**: Baseline, 4, 8, and 24 weeks **Assessment Measures**: • WOMAC (pain, stiffness, and function) • Medication consumption (amount of acetaminophen) **Follow-up**: Baseline and 6 months **Assessment Measures**: • WOMAC (total) • SF-36 (physical function and mental health)	**Experimental Group (EG)**: 2 intra-articular injections (IAI) / platelet-rich plasma (PRP) + prescribed exercises **Control Group (CG)**: Prescribed exercises **Prescribed Exercises**: Quadriceps isometric exercise and hip abductor strengthening; hamstring stretching. Participants were instructed to perform each exercise 3 times per day for 10 s each. After 4 weeks, they were advised to start concentric exercises for the quadriceps, hip abductors, and hip adductors. All participants were instructed to begin exercises 1 week after the IAI. **IAI/PRP**: Two IAIs with a 4-week interval between injections.	**Results**: • Both groups showed improvement at 6 months (T6) in total WOMAC, pain, stiffness, and function. • WOMAC pain scores were better in the EG; however, medication consumption was higher in the EG. • The EG showed better outcomes than the CG for physical function and mental health at T6. • PRP IAI may be effective in reducing pain, stiffness, and improving quality of life (QoL). **Adverse Events**: Transient increase in local pain and swelling. Some patients reported transient knee stiffness, local pelvic pain, and a sensation of swelling. **Exercise Adherence**: Not reported.
**Henriksen et al. (2015)** [34] **RCT** **Full article** **Location**: Denmark **Language**: English	**N**: 100 participants **EG**: • **N**: 50 • **Gender**: 28 F:23 M • **Mean age**: 61.3 years **CG**: • **N**: 50 • **Gender**: 33 F:17 M • **Mean age**: 65.5 years **Condition**: Tibiofemoral osteoarthritis	**Follow-up**: Baseline, 2, 14, and 26 weeks after the IAI **Assessment Measures**: • KOOS (pain) • KOOS (symptoms, daily living, sports function, and knee-related quality of life) • Muscle strength • Walking test • Plasma concentration (IL-6) • MRI (synovitis)	**Experimental Group (EG)**: 1 intra-articular injection (IAI) / corticosteroid (CE) + Exercise Program **Placebo Group (PG)**: 1 intra-articular injection (IAI) / saline + Exercise Program **Exercise Program**: Group exercises, including a 10-minute warm-up on a bicycle, a circuit targeting muscle strength, coordination, and functional knee/hip/trunk exercises with progressive resistance and elastic bands. Conducted twice weekly for 12 weeks. **IAI/CE**: 1 mL of methylprednisolone acetate (Depo-Medrol), 40 mg, dissolved in 4 mL of lidocaine hydrochloride (10 mg/mL). **IAI/Saline**: 1 mL isotonic saline injection mixed with 4 mL of lidocaine hydrochloride (10 mg/mL).	**Statistical Results**: No significant intergroup differences were found for primary or secondary variables. **Specific Findings**: • Isometric hamstring strength (HS) improved more in the EG. • MRI results showed better outcomes in the PG. **Conclusion**: The study found no benefit of corticosteroid treatment compared to placebo when combined with exercises. **Exercise Adherence**: Participants attended an average of 28 out of 36 sessions (78%) in the EG and 29 out of 36 sessions (81%) in the PG. **Adverse Events**: Not reported.
**Saccomanno et al. (2016)** [10] **RCT** **Full article** **Location**: Italy **Language**: English	**N**: 157 participants **G1**: • **N**: 53 • **Gender**: 42 F:11 M • **Mean age**: 62.8 years **G2**: • **N**: 51 • **Gender**: 33 F:18 M • **Mean age**: 61.2 years **G3**: • **N**: 53 • **Gender**: 38 F:15 M • **Mean age**: 61.4 years **Condition**: Mild to moderate osteoarthritis	**Follow-up**: Baseline, 1, 3, and 6 months **Assessment Measures**: • **WOMAC** • **Range of Motion (ROM)**: Flexion	**Group 1 (G1)**: 3 intra-articular injections (IAI) / hyaluronic acid (HA) **Group 2 (G2)**: Exercise program **Group 3 (G3)**: 3 intra-articular injections (IAI) / hyaluronic acid (HA) + Exercise program **Exercise Program**: Participants were divided into 3 subgroups (medial or lateral tibiofemoral OA or patellofemoral OA). A total of 20 sessions per month, 5 times per week (table with exercise descriptions provided). **IAI/HA**: Participants received 3 IAIs (one injection every 2 weeks) of high molecular weight hyaluronic acid (Orthovisc 2 mL; 15 mg/mL; Anika Therapeutics Inc., Bedford, MA).	**WOMAC Pain**: • Group 3 (G3) improved more than Group 1 (G1) at 1 month (T1). • Both Group 2 (G2) and G3 showed improvement at T1 and 6 months (T6). **WOMAC Stiffness and Range of Motion (ROM)**: No statistically significant differences were observed. **WOMAC Function**: No statistically significant intergroup differences were found. G2 experienced a decline in function at T6. **Exercise Adherence**: Not reported. **Adverse Events**: No adverse events were observed.
**Uslu Güvendi et al. (2017)** [32] **RCT** **Full article** **Location**: Turkey **Language**: English	**N**: 57 participants **CSG**: • **N**: 19 • **Gender**: 15 F:2 M • **Mean age**: 62.8 years **PRPG(1)**: • **N**: 19 • **Gender**: 18 F:1 M • **Mean age**: 62.3 years **PRPG(3)**: • **N**: 19 • **Gender**: 13 F:1 M • **Mean age**: 60.4 years **Condition**: Osteoarthritis (Kellgren/Lawrence grade 3) 4o mini	**Follow-up**: Baseline, 2, and 6 months **Assessment Measures**: • Visual Analog Scale (VAS) • WOMAC • Lequesne Index for the Knee • Hospital Anxiety and Depression Scale (HAD) (anxiety and depression)	**Corticosteroid Group (GCE)**: 1 intra-articular injection (IAI) / corticosteroid (CS) **Platelet-Rich Plasma Group (GPRP(1))**: 1 intra-articular injection (IAI) / platelet-rich plasma (PRP) **Platelet-Rich Plasma Group (GPRP(3))**: 3 intra-articular injections (IAI) / platelet-rich plasma (PRP) (administered weekly) **All participants were advised to use ice (if edema occurred)**,** take paracetamol (twice a day as needed)**,** and perform exercises.** **Prescribed Exercises**: Quadriceps strengthening exercises. Participants were instructed to perform 3 sets of 10 repetitions per day. **IAI/CS**: 1 mL containing 6.43 mg of betamethasone dipropionate (equivalent to 5.0 mg of betamethasone) and 2.63 mg of betamethasone sodium phosphate (equivalent to 2.0 mg of betamethasone).	**WOMAC Stiffness**: Higher in GPRP (3) at baseline (T0). **VAS for Movement Pain**: GPRP (3) reported less pain than GCE at T2. GPRP (1) and GPRP (3) showed a greater reduction in pain compared to GCE at T6. **WOMAC Pain**: Lower in GPRP (1) and (3) at T6 compared to GCE. **GCE**: Showed a lesser response compared to PRP (1) or (3). **No statistically significant differences** between GPRP (1) and (3. **HAD**: No changes observed in GCE. **Exercise Adherence**: Not reported. **Adverse Events**: A slight erythema was observed in the knee of one patient in the PRP (1) group. No complaints of edema or local pain were reported.
**Bao et al. (2018)** [11] **RCT** **Full article** **Location**: China **Language**: English	**N**: 60 participants **GA**: • **N**: 20 • **Gender**: 11 females, 9 males • **Mean Age**: 65.3 years **GB**: • **N**: 20 • **Gender**: 10 females, 10 males • **Mean Age**: 66.4 years **GC**: • **N**: 20 • **Gender**: 7 females, 13 males • **Mean Age**: 66.0 years **Condition**: Osteoarthritis (Kellgren/Lawrence grades 2 to 4	**Follow-up**: Baseline, 4, and 8 weeks **Assessment Measures**: • **Visual Analog Scale (VAS)** • **Western Ontario and McMaster Universities Osteoarthritis Index (WOMAC)** • **Short Form-36 (SF-36)** (Physical Function and Mental Health) • **X-ray and MRI** (imaging studies to assess joint structure of the knee)	**GA**: 1 intra-articular injection (IAI) of saline + exercise **GB**: 1 IAI of Botox + exercise **GC**: 5 IAIs of hyaluronic acid + exercise **Exercise Protocol**: • Ice application if pain or edema occurs • 11 exercises focusing on muscle strengthening, balance, and walking • Duration: 30 to 45 min per session, 2 sets of 10 repetitions with progressive load increase • Frequency: 5 times a week for 8 weeks • Exercises were demonstrated in a group setting **IIA/Botox**: 100 U of Botulinum Toxin Type A (Botox; Allergan Inc., Irvine, KY, USA) diluted in 2.5 mL of preservative-free 0.9% saline solution. **IIA/Saline**: 2.5 mL of preservative-free 0.9% saline solution. **IIA/AH**: Sodium hyaluronate (ARTZ, Seikagaku Corporation Takahagi Plant, Ibaraki, Japan) administered once a week for 5 weeks.	**Conclusions**: • The use of Botox and hyaluronic acid (HA) in combination with exercises may help reduce pain and improve function in patients with knee osteoarthritis (OA). • The treatment with Botox + exercises appears to be more effective compared to the other proposed treatments, making it a viable option for these patients. **Results of Imaging Exams**: No statistically significant differences were observed in magnetic resonance imaging (MRI) and X-ray results at T4 and T8. **Results of Assessment Questionnaires**: • At T4: Significant differences in pain scales (VAS) and WOMAC between Groups A and B, and between Groups B and C. • At T8: Significant differences in VAS and WOMAC between Groups A and B, and between Groups B and C. • SF-36 (Physical Functioning and Mental Health): At T4 and T8, significant differences were found between Groups A and B, A and C, and B and C, except for the mental health dimension between Groups A and C at T4. **Adverse Events**: No adverse events were reported during the study. **Exercise Adherence**: No data on exercise adherence were reported.
**Akan et al. (2018)** [31] **RCT** **Full article** **Location**: Turkey **Language**: English	**N**: 62 participants **EG**: • **N**: 31 • **Gender**: 24 F:7 M • **Mean age**: 60.5 years **CG**: • **N**: 31 • **Gender**: 29 F:2 M • **Mean age**: 56.3 years **Condition**: OA (K/L 4)	**Follow-up**: Baseline, 3 and 6 months **Assessment Measures**: • EVA (Visual Analog Scale) • WOMAC (Western Ontario and McMaster Universities Osteoarthritis Index) • SF-36 (Short Form Health Survey) **48 h to 1 year**: effects of IA (intra-articular injections) and history of surgery	**Groups**: • **GE (Exercise Group)**: 3 intra-articular injections of PRP (Platelet-Rich Plasma) + home exercises • **GC (Control Group)**: Home exercises only **Home exercises**: • Knee range of motion exercises • Isometric quadriceps strengthening • Performed 3 times a week, with ice as needed **IIA/PRP Treatment**: 3 intra-articular injections were performed, with 1 injection per week	**Intergroup Differences**: Significant differences in favor of the Exercise Group (GE) at 6 months for all outcomes. **Surgeries Performed in 1 Year**: No patients in the Control Group (GC) underwent arthroplasty, while 1 patient in the GE underwent the procedure. **Adverse Events**: • The most common side effect experienced was a temporary increase in pain. • A total of 13 patients reported temporary increases in pain. • In 6 of these patients, joint swelling in the knee was also reported. **Exercise Adherence**: No data reported.
**Rezasoltani et al. (2020)** [35] **RCT** **Full article** **Location**: Iran **Language**: English	**N**: 120 participants **G1**: • **N**: 30 • **Gender**: 18 F:12 M • **Mean age**: 70.0 years **G2**: • **N**: 30 • **Gender**: 22 F:8 M • **Mean age**: 67.7 years **G3**: • **N**: 30 • **Gender**: 16 F:14 M • **Mean age**: 66.1 years **G4**: • **N**: 30 • **Gender**: 19 F:11 M • **Mean age**: 64.8 years **Condition**: OA (K/L 3 and 4)	**Follow-up**: Baseline, 1 and 4 weeks, and 3 months **Assessment Measures**: • **EVA** • **KOOS**	**G1**: Physiotherapy • 20 min of superficial heat with hot compress + TENS (80 to 100 Hz for 100 to 200 ms) • 5 min of pulsed ultrasound (1 MHz/0.8 to 1.0 W/cm², 50% duty cycle) • Exercise program **G2**: 1 IIA/Botox • 1 IIA with 250 units of Dysport, equivalent to 100 units of botulinum toxin type A (Dysport, Abobotulinumtoxin A; Ipsen, Paris, France), diluted with 5 ml of saline solution • Exercise program **G3**: 3 IIA/AH • 2 ml of hyaluronic acid (Hyalgan; Fidia Farmaceutici, Abano Terme, Italy) administered 3 times a week • Exercise program **G4**: 3 IIA/Dextrose Prolotherapy • 3 IIA, with a 1-month interval, 8 ml of 20% dextrose + 2 ml of 2% lidocaine • Exercise program **Exercise Program**: • Isometric quadriceps strengthening exercises (0°, 45°, and 90°) for 10 s, 10 repetitions, with a 2-second interval • Gastrocnemius and soleus stretching • Each session lasted 30 min.	**EVA**: • G3 was different compared to the other groups, and G2 reduced pain more than G1. • Overall, Botox and Dextrose were the most effective treatments, while HA was the least effective for pain control. **KOOS Pain**: HA was the least effective treatment. **KOOS**: G3 had the least reduction compared to the other groups, and G2 and G4 performed better than physiotherapy for pain, sports, and quality of life. **Adverse Events**: None of the participants experienced or reported serious side effects from the treatments. **Exercise Adherence**: No data reported.
**Sert et al. (2020)** [33] **RCT** **Full article** **Location**: Turkey **Language**: English	**N**: 66 participants **GP**: • **N**: 22 • **Gender**: 18 F:3 M • **Mean age**: 55.7 years **GS**: • **N**: 22 • **Gender**: 20 F:2 M • **Mean age**: 54.4 years **GC**: • **N**: 22 • **Gender**: 17 F:2 M • **Mean age**: 52.0 years **Condition**: OA (K/L 2 or 3)	Follow-up: Baseline, 6, and 18 weeks **Assessment Measures**: • WOMAC (pain, stiffness, and physical function) • EVA (pain and stiffness) • SF-36 (mental and physical health)	• GP: 3 IIA/Dextrose (1 IIA 5 mL of 25% dextrose solution [4 mL of 30% dextrose + 1 mL of 0.9% sodium chloride] and 1 IEA/Dextrose, using the peppering technique and applying a total of 10 mL of 15% dextrose solution [5 mL of 30% dextrose + 2.5 mL of 0.9% sodium chloride + 2.5 mL of 1% lidocaine]) + home exercises • GS: 3 IIA/Saline (2.5 mL of 0.9% sodium chloride + 2.5 mL of 1% lidocaine) and IEA/Saline (5 mL of 0.9% sodium chloride + 5 mL of 1% lidocaine, according to the prolotherapy protocol) + home exercises • GC: Home exercises • Home exercises: IQT stretching and quadriceps isometric strengthening, at the final knee extension, 3 times a week, 3 sets of 10 repetitions • IIA and IEA: 3 IIA and IEA at intervals of 3 weeks (at weeks 0, 3, and 6)	**Pain**: EVA decreased at week 18 in the GP compared to the GS and GC. **Function**: WOMAC function decreased in the GP and GS compared to the GC. **Stiffness**: WOMAC stiffness decreased in the GP compared to the GC in week 18. **Quality of Life (QV)**: No intergroup differences were found for the SF-36 (mental health). In the physical function domain, there was an improvement in the GP compared to the GC in week 18. **Dextrose** had a greater effect on pain, function, and quality of life in patients with knee OA. **Exercise Adherence**: Calculated as the percentage of prescribed sessions attended, adherence was 87.0% for the GP, 86.5% for the GS, and 85% for the GC, with no statistically significant differences between groups. **Adverse Events**: No reports of adverse events.
**Brumini et al. (2023)** [28] **RCT** **Abstract** **Location**: Brazil **Language**: English	**N**: 45 participants **EG**: • **N**: 15 • **Gender**: 14 F: 1 M • **Mean age**: 70.5 years **SG**: • **N**: 15 • **Gender**: 13 F: 2 M • **Mean age**: 71.3 years **GP**: • **N**: 15 • **Gender**: 11 F: 4 M • **Mean age**: 71.9 years **Condition**: OA (K/L 2 or 3)	**Follow-up**: Baseline, 2, 6, and 12 weeks **Assessment Measures**: • **VAS**: pain at rest and during movement • **WOMAC**: pain, stiffness, and physical function • **SF-36**: quality of life • **CJ**: edema • **Functional tests**: SPPB / FTSS / TUGT / 6-minute walk test	**G1**: IIA/CE + PREP - **IIA/CE**: 3 ml (60 mg) of Triamcinolone Hexacetonide (20 mg/ml) + lidocaine **G2**: IIA/Saline + PREP - **IIA/Saline**: 3 ml of saline solution + lidocaine **G3**: IIA/Placebo + PREP - **IIA/Placebo**: no medication **PERP**: • 10 min of warm-up (bicycle) • 4 muscle strengthening exercises (knee flexors and extensors, quadriceps adductors and abductors) • 2 sets of 8 repetitions each • 2 times/week for 3 months	**EVA** (pain at rest and movement), **WOMAC** domains (pain, stiffness, function, and total), **SPPB**, and **1RM** of the 4 muscle groups: no statistically significant intergroup differences were observed. All groups improved over time for all these assessment instruments. **SF-36 -** a difference was found between the groups only for the bodily pain domain (*p* = 0.025). Significant intragroup statistical differences were found in all domains of the SF-36, except for the vitality domain. **Exercise adherence**: The average frequency in the IAI/CE group was 87.75% (21.06 out of 24 sessions), in the IAI/Saline group was 90.83% (21.8 out of 24 sessions), and in the IAI/placebo group was 83.33% (20 out of 24 sessions). **Adverse events**: No adverse events related to the interventions were reported.

RCT = randomized controlled trial; N = number of participants; F = female; M = male; OA = osteoarthritis; EG = experimental group; CG = control group; PG = placebo group; G1 = group 1; G2 = group 2; G3 = group 3; G4 = group 4; CSG = IA group with CS; PRPG (1) = group with 1 IA with PRP; PRPG (3) = group with 3 IA with PRP; GA = group A; GB = group B; GC = group C; DG = IA Dextrose group; SG = IA Saline group; KOOS = consists of 5 subscales: Pain, other symptoms, activities of daily living (ADL), sports and recreation function, and knee-related quality of life; VAS = visual analog scale; WOMAC = Western Ontario and McMaster Universities questionnaire; SF-36 = Short-form 36; HADS = Hospital Anxiety and Depression Scale; FTSS = Five times sit to stand; TTSS = Ten times sit to stand; ROM = range of motion; IAI = intra-articular injection; CS = corticosteroid; HA = hyaluronic acid; PRP = platelet-rich plasma; Ex = exercise; HEP = home exercise program; Saline = saline solution; Botox = botulinum toxin; HABD = hip abductors; HADD = hip adductors; Ham = hamstrings; MS = muscle strengthening

**Table 2:** Risk of bias analysis of the studies included in the systematic review according to the PEdro scale

Studies/ Pedro scale items	1*	2	3	4	5	6	7	8	9	10	11	Total score
1. Partiff 2006 [27]	Y	Y	Y	Y	N	N	N	Y	N	N	Y	5/10
2. Stitik 2007 [29]	Y	Y	N	Y	N	N	Y	Y	Y	Y	Y	7/10
3. Rayegani 2014 [30]	Y	Y	N	Y	N	N	N	Y	N	Y	Y	5/10
4. Henriksen 2015 [34]	Y	Y	Y	Y	Y	Y	Y	Y	Y	Y	Y	10/10
5. Saccomanno 2016 [10]	Y	Y	Y	Y	N	N	Y	Y	N	Y	Y	7/10
6. Guvendi 2018 [32]	Y	Y	N	Y	N	N	Y	Y	N	Y	Y	6/10
7. Bao 2018 [11]	Y	Y	Y	N	Y	N	N	Y	Y	Y	Y	7/10
8. Akan 2018 [31]	Y	Y	Y	Y	N	N	N	Y	N	Y	Y	6/10
9. Rezasoltani 2020 [35]	Y	Y	N	Y	N	N	N	Y	N	Y	Y	5/10
10. Sert 2020 [33]	Y	Y	N	Y	N	N	N	Y	N	Y	Y	6/10
11. Brumini 2023 [28]	Y	Y	Y	Y	Y	Y	Y	N	N	Y	Y	8/10

1: eligibility criteria; 2: random allocation; 3: concealed allocation; 4: baseline comparability; 5: blinded subjects; 6: blinded therapists; 7: blind assessors; 8: adequate follow-up; 9: intention-to-treat analysis; 10: between-group comparisons; 11: point estimates and variability

* does not contribute to the total score

N = No; Y = Yes

The meta-analyses compare intra-articular injections (IAI) of various medications combined with exercise against other IAI treatments and/or placebo, with or without exercise or exercise alone, across short-, medium-, and long-term follow-ups in knee OA patients.

**Fig. 4:**
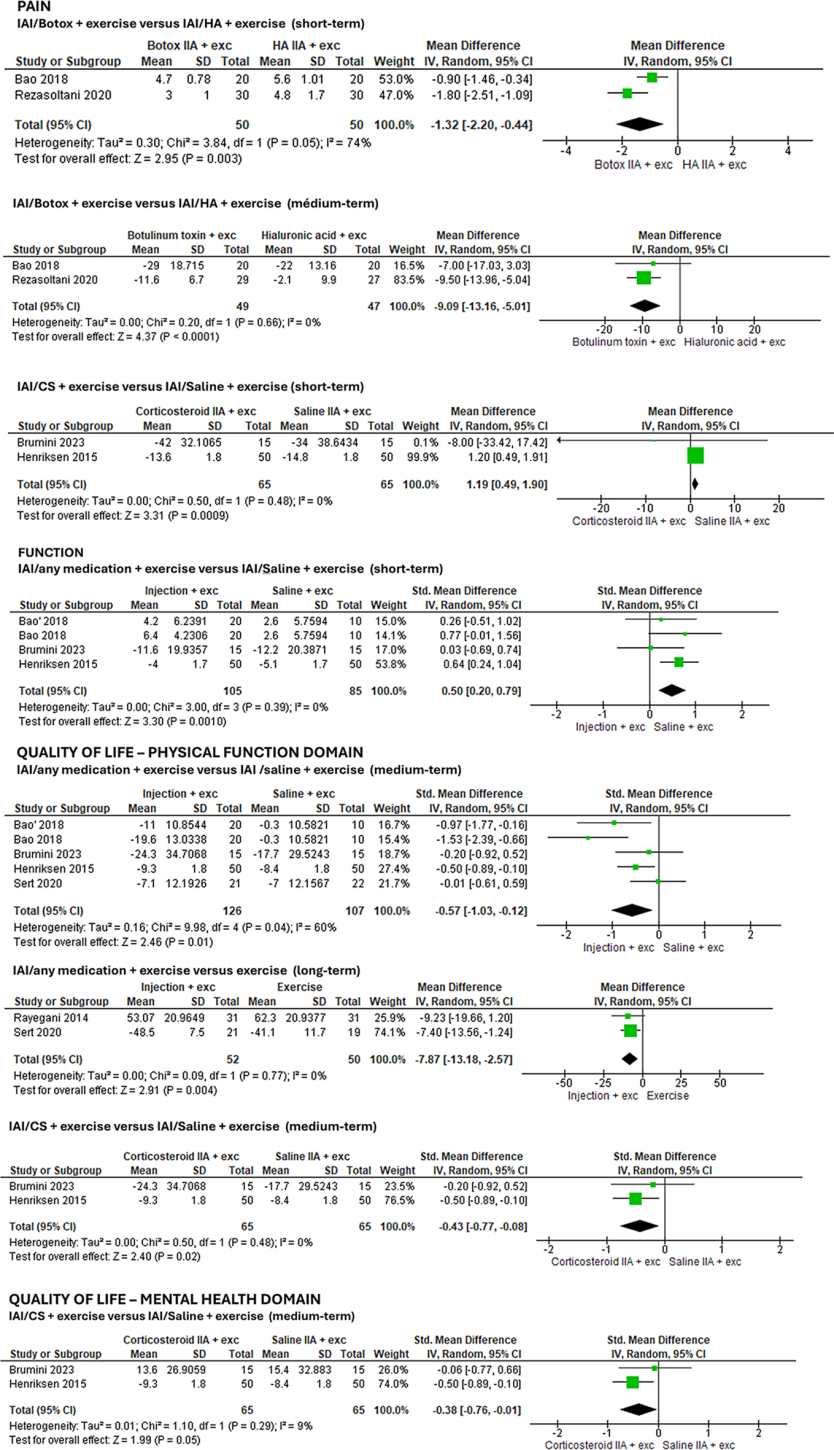
Forest plots of the variables that showed a benefit in the meta-analysis

In Fig. 4, we present the forest plots of the variables that showed a benefit in the meta-analysis. A summary of findings and an assessment of evidence quality using the GRADE approach are presented in Table 3.

**Table 3:** Analysis of the quality of evidence (GRADE) for the variables that showed a benefit in the meta-analysis

Outcomes	№ of participants (studies) Follow-up	Certainty of the evidence (GRADE)	Relative effect (95% CI)	Anticipated absolute effects	
**IAI with any medication + exercise versus IAI/SS + exercise (short term)**				**Risk with Saline injection + exercise** **(short term)**	**Risk difference with Intra-articular injection (any) + exercise**
Function	190 (4 RCTs)	⨁⨁◯◯Low	-	-	SMD **0.5 higher** (0.2 higher to 0.79 higher)
**IAI with any medication + exercise versus IAI/SS + exercise (medium term)**				**Risk with Saline injection + exercise** **(medium term)**	**Risk difference with Intra-articular injection (any) + exercise**
Quality of life - physical function	233 (5 RCTs)	⨁◯◯◯Very low	-	-	SMD **0.57 lower** (1.03 lower to 0.12 lower)
**IAI with any medication + exercise versus exercise (long term)**				**Risk with Exercise (long term)**	**Risk difference with Intra-aticular injection (any) + exercise**
Quality of life - physical function	102 (2 RCTs)	⨁⨁◯◯Low	-	The mean quality of life - physical function was **0**	MD **7.87 lower** (13.18 lower to 2.57 lower)
**IAI/CE + exercise versus IAI/SS + exercise (medium term)**				**Risk with Saline + exercise (medium term)**	**Risk difference with Corticosteroid + exercise**
Pain	130 (2 RCTs)	⨁⨁◯◯Low	-	The mean pain was **0**	MD **1.19 higher** (0.49 higher to 1.9 higher)
Quality of life - physical function	130 (2 RCTs)	⨁⨁◯◯Low	-	-	SMD **0.43 lower** (0.77 lower to 0.08 lower)
Quality of life - mental health	130 (2 RCTs)	⨁⨁◯◯Low	-	-	SMD **0.38 lower** (0.76 lower to 0.01 lower)
**IAI/Botox + exercise versus IIA/HA + exercise (short term)**				**Risk with Hialuronic acid + exercise** **(short term)**	**Risk difference with Botulinum toxin + exercise**
Pain	100 (2 RCTs)	⨁◯◯◯Very low	-	The mean pain was **0**	MD **1.32 lower** (2.2 lower to 0.44 lower)
**IAI/Botox + exercise versus IAI/HA + exercise (long term)**				**Risk with Hialuronic acid + exercise** **(medium term)**	**Risk difference with Botulinum toxin + exercise**
Pain	96 (2 RCTs)	⨁⨁◯◯Low	-	The mean pain was **0**	MD **9.09 lower** (13.16 lower to 5.01 lower)

**CI**: confidence interval; **MD**: mean difference; IIA = injeção intra-articular; IIA/SS = injeção intra-articular com solução salina; IIA/CE = injeção intra-articular com corticoesteroide; IIA/AH = injeção intra-articular com ácido hialurônico; IIA/Botox = injeção intra-articular com toxina botulínica

**GRADE Working Group grades of evidence**

**High certainty**: we are very confident that the true effect lies close to that of the estimate of the effect

**Moderate certainty**: we are moderately confident in the effect estimate: the true effect is likely to be close to the estimate of the effect, but there is a possibility that it is substantially different

**Low certainty**: our confidence in the effect estimate is limited: the true effect may be substantially different from the estimate of the effect

**Very low certainty**: we have very little confidence in the effect estimate: the true effect is likely to be substantially different from the estimate of effect

Additional analyses of comparisons without statistically significant differences are available in Supplementary Figs. 1, 2, and 3. Supplementary Table 2 provides an assessment of evidence quality using the GRADE approach for the variables that showed no benefit in meta-analysis.

## Discussion

The findings of this systematic review and meta-analysis provide nuanced insights into the combined effects of intra-articular injections (IAI) and exercise on knee osteoarthritis (OA). While the results demonstrate certain benefits in pain reduction, functional improvement, and quality of life, the evidence is classified as low to very low quality, necessitating a critical examination of the reasons behind these outcomes.

In the short term, Botulinum toxin IAI combined with exercise resulted in greater pain relief compared to Hyaluronic Acid (HA) IAI. This difference may be attributed to the distinct mechanisms of action of Botulinum toxin, which inhibits acetylcholine release at the neuromuscular junction, potentially reducing muscle overactivity and pain [11, 35]. Conversely, HA primarily provides mechanical lubrication and viscoelastic properties, which may result in a slower onset of pain relief [10]. These findings suggest that the choice of IAI medication should consider the underlying pathophysiology of the patient’s symptoms. Future research should explore whether stratifying patients based on clinical characteristics, such as muscle spasticity or synovial inflammation, could help optimize treatment outcomes.

For functional outcomes, Saline IAI combined with exercise appeared to be more effective than IAI with any medication in the short term. This result may reflect the benefits of exercise itself, as saline injections likely serve as a placebo [5]. Exercise, particularly progressive resistance training, is well-documented to enhance muscle strengthening and joint stabilization, which may account for the observed improvements [34]. The lack of significant differences between saline and medicated IAI groups underscores the importance of non-pharmacological interventions, further emphasizing exercise as a cornerstone of knee OA management [3]. Future studies should focus on refining exercise protocols to enhance adherence and effectiveness, potentially incorporating tele-rehabilitation or wearable technology to support home-based programs [6].

In the medium term, Corticosteroid IAI combined with exercise was associated with improvements in quality of life, particularly in physical and mental health domains [7]. Corticosteroids are widely recognized for their anti-inflammatory properties, which may complement the benefits of exercise in reducing joint inflammation and pain [1]. However, the short-lived effects of corticosteroids and their potential adverse outcomes, such as cartilage degradation with repeated administration, necessitate careful consideration [2]. Combining corticosteroid IAIs with long-term, structured exercise programs may help mitigate these risks while maintaining therapeutic benefits. This approach aligns with current guidelines that advocate for multimodal, personalized treatment strategies [4].

The long-term benefits observed for IAI combined with exercise, particularly in the domain of physical function, suggest that IAIs may play a supportive role in prolonging the effects of exercise [36]. However, limited adherence to home-based exercise programs in the included studies likely attenuates the potential benefits. These adherence challenges highlight the need for strategies to encourage consistent patient engagement, such as supervised group sessions, motivational interviewing, or digital health interventions [27].

One notable limitation across the studies was the heterogeneity in exercise regimens and IAI protocols, including variations in dosage, frequency, and types of medications [15]. Standardizing these interventions in future trials would improve comparability and facilitate clearer clinical guidelines. Additionally, the low quality of evidence, as assessed by GRADE criteria, highlights the need for rigorously designed, high-quality randomized controlled trials featuring larger sample sizes and robust blinding procedures [37].

## Conclusion

The findings of this review suggest that the combination of intra-articular injections (IAI) and exercise may offer benefits for managing knee osteoarthritis (OA). Specifically:

- **Pain Outcome**: Botulinum toxin IAI combined with exercise was more effective than hyaluronic acid IAI in the short and medium term. Additionally, Saline IAI demonstrated greater medium-term pain relief compared to Corticosteroid IAI.
- **Functional Outcome**: In the short term, Saline IAI combined with exercise was linked to superior functional outcomes compared to IAI with any medication, highlighting the significant contribution of exercise to functional improvements.
- **Quality of Life**: Corticosteroid IAI combined with exercise improved quality of life in the medium term, particularly in the physical and mental health domains. In the long term, IAI combined with exercise was superior to exercise alone for enhancing physical function.

While the evidence is classified as low to very low quality, these findings highlight the importance of personalized treatment strategies that combine pharmacological and non-pharmacological interventions. Both IAIs and exercise are considered safe for knee OA management, although repeated corticosteroid use should be approached with caution due to potential adverse effects. Future research should focus on standardizing protocols, enhancing adherence strategies, and tailoring treatments to meet patient-specific needs in order to optimize outcomes.

## Electronic supplementary material

Below is the link to the electronic supplementary material.

Supplementary Material 1

Supplementary Material 2

Supplementary Material 3

Supplementary Material 4

Supplementary Material 5

## Data Availability

No datasets were generated or analysed during the current study.
