# Supplementary material for: Exercises plus intra-articular injection for knee osteoarthritis: a systematic review with meta-analysis
Source: Adv Rheumatol. 2025 Dec 10;66:5. doi: 10.1186/s42358-025-00458-3 (PMC13472110; doi:10.1186/s42358-025-00458-3)
Supplement: Supplementary file 1 [file 42358_2025_458_MOESM1_ESM.docx]

**Appendix 1. Search Strategies in Databases: PubMed (MEDLINE), EMBASE, CENTRAL/Cochrane, CINAHL, and PEDro**

| **Database** | **Strategies** | **Regiters** |
| --- | --- | --- |
| **PubMed / MEDLINE** | #1 "osteoarthritis"[MeSH Terms] OR "osteoarthritis"[All Fields] OR "osteoarthritides"[All Fields] OR ("osteoarthritis"[MeSH Terms] OR "osteoarthritis"[All Fields] OR "osteoarthritides"[All Fields]) OR ("osteoarthritis"[MeSH Terms] OR "osteoarthritis"[All Fields] OR "osteoarthrosis"[All Fields]) OR ("osteoarthritis"[MeSH Terms] OR "osteoarthritis"[All Fields] OR "osteoarthroses"[All Fields]) OR ("osteoarthritis"[MeSH Terms] OR "osteoarthritis"[All Fields] OR ("arthritis"[All Fields] AND "degenerative"[All Fields]) OR "arthritis degenerative"[All Fields]) OR ("osteoarthritis"[MeSH Terms] OR "osteoarthritis"[All Fields] OR ("arthritides"[All Fields] AND "degenerative"[All Fields])) OR ("osteoarthritis"[MeSH Terms] OR "osteoarthritis"[All Fields] OR ("degenerative"[All Fields] AND "arthritides"[All Fields]) OR "degenerative arthritides"[All Fields]) OR ("osteoarthritis"[MeSH Terms] OR "osteoarthritis"[All Fields] OR ("degenerative"[All Fields] AND "arthritis"[All Fields]) OR "degenerative arthritis"[All Fields]) OR ("joint diseases"[MeSH Terms] OR ("joint"[All Fields] AND "diseases"[All Fields]) OR "joint diseases"[All Fields] OR "arthrosis"[All Fields]) OR ("arthrosic"[All Fields] OR "osteoarthritis"[MeSH Terms] OR "osteoarthritis"[All Fields] OR "arthroses"[All Fields]) OR ("osteoarthritis"[MeSH Terms] OR "osteoarthritis"[All Fields] OR ("osteoarthrosis"[All Fields] AND "deformans"[All Fields]) OR "osteoarthrosis deformans"[All Fields]) OR ("osteoarthritis, knee"[MeSH Terms] OR ("osteoarthritis"[All Fields] AND "knee"[All Fields]) OR "knee osteoarthritis"[All Fields] OR ("knee"[All Fields] AND "osteoarthritides"[All Fields])) OR ("osteoarthritis, knee"[MeSH Terms] OR ("osteoarthritis"[All Fields] AND "knee"[All Fields]) OR "knee osteoarthritis"[All Fields] OR ("osteoarthritis"[All Fields] AND "knee"[All Fields]) OR "osteoarthritis of knee"[All Fields]) OR ("osteoarthritis, knee"[MeSH Terms] OR ("osteoarthritis"[All Fields] AND "knee"[All Fields]) OR "knee osteoarthritis"[All Fields] OR ("osteoarthritis"[All Fields] AND "knee"[All Fields]) OR "osteoarthritis of the knee"[All Fields]) OR (("arthritis"[MeSH Terms] OR "arthritis"[All Fields] OR "arthritides"[All Fields] OR "polyarthritides"[All Fields]) AND "noninflammatory"[All Fields]) OR ("osteoarthritis"[MeSH Terms] OR "osteoarthritis"[All Fields] OR ("degenerative"[All Fields] AND "joint"[All Fields] AND "disease"[All Fields]) OR "degenerative joint disease"[All Fields]) OR "osteo-arthritis"[All Fields] OR "osteo-arthrosis"[All Fields] OR (("primaries"[All Fields] OR "primary"[All Fields]) AND ("osteoarthritis"[MeSH Terms] OR  "osteoarthritis"[All Fields] OR "osteoarthritides"[All Fields])) OR (("rheumatoid"[All Fields] OR "rheumatoids"[All Fields]) AND ("joint diseases"[MeSH Terms] OR ("joint"[All Fields] AND "diseases"[All Fields]) OR "joint diseases"[All Fields] OR "arthrosis"[All Fields])) OR ("osteoarthritis, knee"[MeSH Terms] OR ("osteoarthritis"[All Fields] AND "knee"[All Fields]) OR "knee osteoarthritis"[All Fields] OR ("knee"[All Fields] AND "osteoarthritis"[All Fields]))  #2 "knee"[MeSH Terms] OR "knee"[All Fields] OR "knee joint"[MeSH Terms] OR ("knee"[All Fields] AND "joint"[All Fields]) OR "knee joint"[All Fields] OR ("joint*"[All Fields] AND ("knee"[MeSH Terms] OR "knee"[All Fields] OR "knee joint"[MeSH Terms] OR ("knee"[All Fields] AND "joint"[All Fields]) OR "knee joint"[All Fields])) OR ("knee joint"[MeSH Terms] OR ("knee"[All Fields] AND "joint"[All Fields]) OR "knee joint"[All Fields] OR ("knee"[All Fields] AND "joints"[All Fields]) OR "knee joints"[All Fields]) OR "knee*"[All Fields] OR ("knee joint"[MeSH Terms] OR ("knee"[All Fields] AND "joint"[All Fields]) OR "knee joint"[All Fields] OR ("superior"[All Fields] AND "tibiofibular"[All Fields] AND "joint"[All Fields]) OR "superior tibiofibular joint"[All Fields]) OR ("knee joint"[MeSH Terms] OR ("knee"[All Fields] AND "joint"[All Fields]) OR "knee joint"[All Fields] OR ("joint"[All Fields] AND "superior"[All Fields] AND "tibiofibular"[All Fields])) OR ("knee joint"[MeSH Terms] OR ("knee"[All Fields] AND "joint"[All Fields]) OR "knee joint"[All Fields] OR ("joints"[All Fields] AND "superior"[All Fields] AND "tibiofibular"[All Fields])) OR ("knee joint"[MeSH Terms] OR ("knee"[All Fields] AND "joint"[All Fields]) OR "knee joint"[All Fields] OR ("superior"[All Fields] AND "tibiofibular"[All Fields] AND "joints"[All Fields]) OR "superior tibiofibular joints"[All Fields]) OR ("knee joint"[MeSH Terms] OR ("knee"[All Fields] AND "joint"[All Fields]) OR "knee joint"[All Fields] OR ("tibiofibular"[All Fields] AND "joint"[All Fields] AND "superior"[All Fields]) OR "tibiofibular joint superior"[All Fields]) OR ("knee joint"[MeSH Terms] OR ("knee"[All Fields] AND "joint"[All Fields]) OR "knee joint"[All Fields] OR ("tibiofibular"[All Fields] AND "joints"[All Fields] AND "superior"[All Fields]))  #3 "exercise"[MeSH Terms] OR "exercise"[All Fields] OR "exercises"[All Fields] OR "exercise therapy"[MeSH Terms] OR ("exercise"[All Fields] AND "therapy"[All Fields]) OR "exercise therapy"[All Fields] OR "exercise s"[All Fields] OR "exercised"[All Fields] OR "exerciser"[All Fields] OR "exercisers"[All Fields] OR "exercising"[All Fields] OR "exercise*"[All Fields] OR ("exercise"[MeSH Terms] OR "exercise"[All Fields] OR ("physical"[All Fields] AND "activity"[All Fields]) OR "physical activity"[All Fields]) OR ("exercise"[MeSH Terms] OR "exercise"[All Fields] OR ("activities"[All Fields] AND "physical"[All Fields]) OR "activities physical"[All Fields]) OR ("exercise"[MeSH Terms] OR "exercise"[All Fields] OR ("activity"[All Fields] AND "physical"[All Fields]) OR "activity physical"[All Fields]) OR ("exercise"[MeSH Terms] OR "exercise"[All Fields] OR ("physical"[All Fields] AND "activities"[All Fields]) OR "physical activities"[All Fields]) OR ("exercise"[MeSH Terms] OR "exercise"[All Fields] OR ("exercise"[All Fields] AND "physical"[All Fields]) OR "exercise physical"[All Fields]) OR ("exercise"[MeSH Terms] OR "exercise"[All Fields] OR ("exercises"[All Fields] AND "physical"[All Fields]) OR "exercises physical"[All Fields]) OR ("exercise"[MeSH Terms] OR "exercise"[All Fields] OR ("physical"[All Fields] AND "exercise"[All Fields]) OR "physical exercise"[All Fields]) OR ("exercise"[MeSH Terms] OR "exercise"[All Fields] OR ("physical"[All Fields] AND "exercises"[All Fields]) OR "physical exercises"[All Fields]) OR ("exercise"[MeSH Terms] OR "exercise"[All Fields] OR ("acute"[All Fields] AND "exercise"[All Fields]) OR "acute exercise"[All Fields]) OR ("exercise"[MeSH Terms] OR "exercise"[All Fields] OR ("acute"[All Fields] AND "exercises"[All Fields]) OR "acute exercises"[All Fields]) OR ("exercise"[MeSH Terms] OR "exercise"[All Fields] OR ("exercise"[All Fields] AND "acute"[All Fields]) OR "exercise acute"[All Fields]) OR ("exercise"[MeSH Terms] OR "exercise"[All Fields] OR ("exercises"[All Fields] AND "acute"[All Fields]) OR "exercises acute"[All Fields]) OR ("exercise"[MeSH Terms] OR "exercise"[All Fields] OR ("exercise"[All Fields] AND "isometric"[All Fields]) OR "exercise isometric"[All Fields]) OR ("exercise"[MeSH Terms] OR "exercise"[All Fields] OR ("isometric"[All Fields] AND "exercise"[All Fields]) OR "isometric exercise"[All Fields]) OR ("exercise"[MeSH Terms] OR "exercise"[All Fields] OR ("exercises"[All Fields] AND "isometric"[All Fields]) OR "exercises isometric"[All Fields]) OR ("exercise"[MeSH Terms] OR "exercise"[All Fields] OR ("isometric"[All Fields] AND "exercises"[All Fields]) OR "isometric exercises"[All Fields]) OR ("exercise"[MeSH Terms] OR "exercise"[All Fields] OR ("exercise"[All Fields] AND "aerobic"[All Fields]) OR "exercise aerobic"[All Fields]) OR ("exercise"[MeSH Terms] OR "exercise"[All Fields] OR ("aerobic"[All Fields] AND "exercise"[All Fields]) OR "aerobic exercise"[All Fields]) OR ("exercise"[MeSH Terms] OR "exercise"[All Fields] OR ("aerobic"[All Fields] AND "exercises"[All Fields]) OR "aerobic exercises"[All Fields]) OR ("exercise"[MeSH Terms] OR "exercise"[All Fields] OR ("exercises"[All Fields] AND "aerobic"[All Fields]) OR "exercises aerobic"[All Fields]) OR  ("exercise"[MeSH Terms] OR "exercise"[All Fields] OR ("exercise"[All Fields] AND "training"[All Fields]) OR "exercise training"[All Fields]) OR ("exercise"[MeSH Terms] OR "exercise"[All Fields] OR ("exercise"[All Fields] AND "trainings"[All Fields]) OR "exercise trainings"[All Fields]) OR ("exercise"[MeSH Terms] OR "exercise"[All Fields] OR ("training"[All Fields] AND "exercise"[All Fields]) OR "training exercise"[All Fields]) OR ("exercise"[MeSH Terms] OR "exercise"[All Fields] OR ("trainings"[All Fields] AND "exercise"[All Fields])) OR ("exercise therapy"[MeSH Terms] OR ("exercise"[All Fields] AND "therapy"[All Fields]) OR "exercise therapy"[All Fields] OR ("exercise"[All Fields] AND "therapies"[All Fields]) OR "exercise therapies"[All Fields]) OR ("exercise therapy"[MeSH Terms] OR ("exercise"[All Fields] AND "therapy"[All Fields]) OR "exercise therapy"[All Fields] OR ("exercise"[All Fields] AND "rehabilitation"[All Fields]) OR "exercise rehabilitation"[All Fields]) OR ("exercise therapy"[MeSH Terms] OR ("exercise"[All Fields] AND "therapy"[All Fields]) OR "exercise therapy"[All Fields] OR ("exercises"[All Fields] AND "rehabilitation"[All Fields]) OR "exercises rehabilitation"[All Fields]) OR ("resistance training"[MeSH Terms] OR ("resistance"[All Fields] AND "training"[All Fields]) OR "resistance training"[All Fields] OR ("training"[All Fields] AND "resistance"[All Fields]) OR "training resistance"[All Fields]) OR ("resistance training"[MeSH Terms] OR ("resistance"[All Fields] AND "training"[All Fields]) OR "resistance training"[All Fields] OR ("strength"[All Fields] AND "training"[All Fields]) OR "strength training"[All Fields]) OR ("muscle stretching exercises"[MeSH Terms] OR ("muscle"[All Fields] AND "stretching"[All Fields] AND "exercises"[All Fields]) OR "muscle stretching exercises"[All Fields] OR ("exercise"[All Fields] AND "muscle"[All Fields] AND "stretching"[All Fields]) OR "exercise muscle stretching"[All Fields]) OR (("muscle s"[All Fields] OR "muscles"[MeSH Terms] OR "muscles"[All Fields] OR "muscle"[All Fields]) AND ("stretch"[All Fields] OR "stretched"[All Fields] OR "stretches"[All Fields] OR "stretching"[All Fields] OR "stretchings"[All Fields]) AND "exercise*"[All Fields]) OR ("exercise movement techniques"[MeSH Terms] OR ("exercise"[All Fields] AND "movement"[All Fields] AND "techniques"[All Fields]) OR "exercise movement techniques"[All Fields] OR ("exercise"[All Fields] AND "movement"[All Fields] AND "technics"[All Fields]) OR "exercise movement technics"[All Fields]) OR ("exercise movement techniques"[MeSH Terms] OR ("exercise"[All Fields] AND "movement"[All Fields] AND "techniques"[All Fields]) OR "exercise movement techniques"[All Fields] OR ("pilates"[All Fields] AND "based"[All Fields] AND "exercises"[All Fields]) OR "pilates based exercises"[All Fields]) OR (("stretch"[All Fields] OR "stretched"[All Fields] OR "stretches"[All Fields] OR "stretching"[All Fields] OR "stretchings"[All Fields]) AND ("exercise"[MeSH Terms] OR "exercise"[All Fields] OR "exercises"[All Fields] OR "exercise therapy"[MeSH Terms] OR ("exercise"[All Fields] AND "therapy"[All Fields]) OR "exercise therapy"[All Fields] OR "exercise s"[All Fields] OR "exercised"[All Fields] OR "exerciser"[All Fields] OR "exercisers"[All Fields] OR "exercising"[All Fields]))  #4 "injections, intra articular"[MeSH Terms] OR ("injections"[All Fields] AND "intra articular"[All Fields]) OR "intra-articular injections"[All Fields] OR ("injections"[All Fields] AND "intra"[All Fields] AND "articular"[All Fields]) OR "injections intra articular"[All Fields] OR ("injections, intra articular"[MeSH Terms] OR ("injections"[All Fields] AND "intra articular"[All Fields]) OR "intra-articular injections"[All Fields] OR ("intraarticular"[All Fields] AND "injections"[All Fields]) OR "intraarticular injections"[All Fields]) OR ("injections, intra articular"[MeSH Terms] OR ("injections"[All Fields] AND "intra articular"[All Fields]) OR "intra-articular injections"[All Fields] OR ("injections"[All Fields] AND "intraarticular"[All Fields]) OR "injections intraarticular"[All Fields]) OR ("injections, intra articular"[MeSH Terms] OR ("injections"[All Fields] AND "intra articular"[All Fields]) OR "intra-articular injections"[All Fields] OR ("injection"[All Fields] AND "intraarticular"[All Fields]) OR "injection intraarticular"[All Fields]) OR ("intraarticular"[All Fields] AND "drug"[All Fields] AND ("administrable"[All Fields] OR "administrate"[All Fields] OR "administrated"[All Fields] OR "administrating"[All Fields] OR "administrations"[All Fields] OR "administred"[All Fields] OR "administrered"[All Fields] OR "organization and administration"[MeSH Terms] OR ("organization"[All Fields] AND "administration"[All Fields]) OR "organization and administration"[All Fields] OR "administration"[All Fields])) OR ("injections, intra articular"[MeSH Terms] OR ("injections"[All Fields] AND "intra articular"[All Fields]) OR "intra-articular injections"[All Fields] OR ("intraarticular"[All Fields] AND "injection"[All Fields]) OR "intraarticular injection"[All Fields]) OR ("injections, intra articular"[MeSH Terms] OR ("injections"[All Fields] AND "intra articular"[All Fields]) OR "intra-articular injections"[All Fields] OR ("intra"[All Fields] AND "articular"[All Fields] AND "injections"[All Fields]) OR "intra articular injections"[All Fields]) OR ("injections, intra articular"[MeSH Terms] OR ("injections"[All Fields] AND "intra articular"[All Fields]) OR "intra-articular injections"[All Fields] OR ("injection"[All Fields] AND "intra"[All Fields] AND "articular"[All Fields]) OR "injection intra articular"[All Fields]) OR ("injections, intra articular"[MeSH Terms]  OR ("injections"[All Fields] AND "intra articular"[All Fields]) OR "intra-articular injections"[All Fields] OR ("intra"[All Fields] AND "articular"[All Fields] AND "injections"[All Fields]) OR "intra articular injections"[All Fields]) OR "intra"[All Fields]  #5 "randomized controlled trial"[Publication Type] OR "randomized controlled trials as topic"[MeSH Terms] OR "randomized controlled trial"[All Fields] OR "randomised controlled trial"[All Fields] OR ("controlled clinical trial"[Publication Type] OR "controlled clinical trials as topic"[MeSH Terms] OR "controlled clinical trial"[All Fields]) OR ("comparative study"[Publication Type] OR "comparative study"[All Fields]) OR ("clinical trial"[Publication Type] OR "clinical trials as topic"[MeSH Terms] OR "clinical trial"[All Fields]) OR ("random allocation"[MeSH Terms] OR ("random"[All Fields] AND "allocation"[All Fields]) OR "random allocation"[All Fields] OR "random"[All Fields] OR "randomization"[All Fields] OR "randomized"[All Fields] OR "randomisation"[All Fields] OR "randomisations"[All Fields] OR "randomise"[All Fields] OR "randomised"[All Fields] OR "randomising"[All Fields] OR "randomizations"[All Fields] OR "randomize"[All Fields] OR "randomizes"[All Fields] OR "randomizing"[All Fields] OR "randomness"[All Fields] OR "randoms"[All Fields]) OR ("placeboes"[All Fields] OR "placebos"[MeSH Terms] OR "placebos"[All Fields] OR "placebo"[All Fields]) OR ("drug therapy"[MeSH Subheading] OR ("drug"[All Fields] AND "therapy"[All Fields]) OR "drug therapy"[All Fields] OR "drug therapy"[MeSH Terms] OR ("drug"[All Fields] AND "therapy"[All Fields])) OR "randomly"[All Fields] OR ("clinical trials as topic"[MeSH Terms] OR ("clinical"[All Fields] AND "trials"[All Fields] AND "topic"[All Fields]) OR "clinical trials as topic"[All Fields] OR "trial"[All Fields] OR "trial s"[All Fields] OR "trialed"[All Fields] OR "trialing"[All Fields] OR "trials"[All Fields]) OR ("groups"[All Fields] OR "grouped"[All Fields] OR "grouping"[All Fields] OR "groupings"[All Fields] OR "groups"[All Fields] OR "groups s"[All Fields] OR "population groups"[MeSH Terms] OR ("population"[All Fields] AND "groups"[All Fields]) OR "population groups"[All Fields] OR "group"[All Fields])  #1 AND #2 AND #3 AND #4 AND #5 | **311** |
| **Embase** | #1 ('osteoarthritis'/exp OR 'osteoarthritis' OR osteoarthritides OR 'osteoarthrosis' OR 'osteoarthrosis'/exp OR osteoarthrosis OR osteoarthroses OR 'arthritis degenerative'/exp OR 'arthritis degenerative' OR 'arthritides degenerative' OR 'degenerative arthritides' OR 'degenerative arthritis'/exp OR 'degenerative arthritis' OR 'arthrosis' OR 'arthrosis'/exp OR arthrosis OR arthroses OR 'osteoarthrosis deformans' OR 'knee osteoarthritides' OR 'osteoarthritis of knee' OR 'osteoarthritis of the knee' OR 'arthritis noninflammatory'/exp OR 'arthritis noninflammatory' OR 'degenerative joint disease'/exp OR 'degenerative joint disease' OR 'osteo-arthritis'/exp OR 'osteo-arthritis' OR 'osteo-arthrosis'/exp OR 'osteo-arthrosis' OR 'primary osteoarthritis'/exp OR 'primary osteoarthritis' OR 'rheumatoid arthrosis'/exp OR 'rheumatoid arthrosis' OR 'knee osteoarthritis'/exp OR 'knee osteoarthritis') AND [embase]/lim  #2 ('knee'/exp OR 'knee' OR 'joint$ knee' OR 'knee joints' OR knee$ OR 'superior tibiofibular joint' OR 'joint superior tibiofibular' OR 'joints superior tibiofibular' OR 'superior tibiofibular joints' OR 'tibiofibular joint superior' OR 'tibiofibular joints superior') AND [embase]/lim  #3 ('exercise'/exp OR 'exercise' OR exercise$ OR 'physical activity'/exp OR 'physical activity' OR 'activities physical' OR 'activity physical'/exp OR 'activity physical' OR 'physical activities' OR 'exercise physical' OR 'exercises physical' OR 'physical exercise'/exp OR 'physical exercise' OR 'physical exercises' OR 'acute exercise'/exp OR 'acute exercise' OR 'acute exercises' OR 'exercise acute' OR 'exercises acute' OR 'exercise isometric'/exp OR 'exercise isometric' OR 'isometric exercise'/exp OR  'isometric exercise' OR 'exercises isometric' OR 'isometric exercises' OR 'exercise aerobic'/exp OR 'exercise aerobic' OR 'aerobic exercise'/exp OR 'aerobic exercise' OR 'aerobic exercises' OR 'exercises aerobic' OR 'exercise training'/exp OR 'exercise training' OR 'exercise trainings' OR 'training exercise' OR 'trainings exercise' OR 'exercise therapies' OR 'exercise rehabilitation' OR 'exercises rehabilitation' OR 'training resistance' OR 'strength training'/exp OR 'strength training' OR 'exercise muscle stretching' OR ‘muscle stretching exercise$’ OR 'exercise movement technics' OR 'pilates-based exercises' OR 'stretching exercise'/exp OR 'stretching exercise') AND [embase]/lim  #4 ('injections intra articular'/exp OR 'injections intra articular' OR 'intraarticular injections' OR 'injections intraarticular' OR 'injection intraarticular'/exp OR 'injection intraarticular' OR 'intraarticular drug administration'/exp OR 'intraarticular drug administration' OR 'intraarticular injection'/exp OR 'intraarticular injection' OR 'intra-articular injections'/exp OR 'intra-articular injections' OR 'injection intra-articular' OR 'intra articular injections'/exp OR 'intra articular injections' OR 'intra-articular injection'/exp OR 'intra-articular injection') AND [embase]/lim  #5('clinical article' OR 'clinical study'/exp OR 'clinical trial' OR 'controlled study' OR 'randomized controlled trial' OR 'major clinical study' OR 'double blind procedure' OR 'multicenter study' OR 'single blind procedure' OR 'phase 3 clinical trial' OR 'phase 4 clinical trial' OR 'crossover procedure' OR 'placebo' OR allocat* OR assign* OR compar* OR control* OR cross*over OR factorial* OR follow*up OR placebo* OR prospectiv* OR random* OR 'trial' OR versus OR vs OR singl* OR doubl* OR trebl* OR tripl* OR blind* OR mask*) NOT animal*  #1 AND #2 and #3 AND #4 AND #5 | **455** |
| **CENTRAL / COCHRANE** | #1 osteoarthritis OR osteoarthritides OR osteoarthrosis OR osteoarthroses OR arthritis degenerative OR arthritides degenerative OR degenerative arthritides OR degenerative arthritis OR arthrosis OR arthroses OR osteoarthrosis deformans OR knee osteoarthritides OR osteoarthritis of knee OR osteoarthritis of the knee OR arthritis noninflammatory OR degenerative joint disease OR osteo-arthritis OR osteo-arthrosis OR primary osteoarthritis OR rheumatoid arthrosis OR knee osteoarthritis  #2 knee OR joint* knee OR knee joints OR knee* OR superior tibiofibular joint OR joint superior tibiofibular OR joints superior tibiofibular OR superior tibiofibular joints OR tibiofibular joint superior OR tibiofibular joints superior  #3 exercise OR exercise* OR physical activity OR activities physical OR activity physical OR physical activities OR exercise physical OR exercises physical OR physical exercise OR physical exercises OR acute exercise OR acute exercises OR exercise acute OR exercises acute OR exercise isometric OR isometric exercise OR exercises isometric OR isometric exercises OR exercise aerobic OR aerobic exercise OR aerobic exercises OR exercises aerobic OR exercise training OR exercise trainings OR training exercise OR trainings exercise OR exercise therapies OR exercise rehabilitation OR exercises rehabilitation OR training resistance OR strength training OR exercise muscle stretching OR muscle stretching exercise* OR exercise movement technics OR pilates-based exercises OR stretching exercise  #4 injections intra articular OR intraarticular injections OR injections intraarticular OR injection intraarticular OR intraarticular drug administration OR intraarticular injection OR intra-articular injections OR injection intra-articular OR intra articular injections OR intra-articular injection  #1 AND #2 AND #3 AND #4 AND Limits: TRIALS | **217** |
| **CINAHL** | #1 osteoarthritis OR osteoarthritides OR osteoarthrosis OR osteoarthroses OR arthritis degenerative OR arthritides degenerative OR degenerative arthritides OR degenerative arthritis OR arthrosis OR arthroses OR osteoarthrosis deformans OR knee osteoarthritides OR osteoarthritis of knee OR osteoarthritis of the  knee OR arthritis noninflammatory OR degenerative joint disease OR osteo-arthritis OR osteo-arthrosis OR primary osteoarthritis OR rheumatoid arthrosis OR knee osteoarthritis  #2 knee OR joint* knee OR knee joints OR knee* OR superior tibiofibular joint OR joint superior tibiofibular OR joints superior tibiofibular OR superior tibiofibular joints OR tibiofibular joint superior OR tibiofibular joints superior  #3 exercise OR exercise* OR physical activity OR activities physical OR activity physical OR physical activities OR exercise physical OR exercises physical OR physical exercise OR physical exercises OR acute exercise OR acute exercises OR exercise acute OR exercises acute OR exercise isometric OR isometric exercise OR exercises isometric OR isometric exercises OR exercise aerobic OR aerobic exercise OR aerobic exercises OR exercises aerobic OR exercise training OR exercise trainings OR training exercise OR trainings exercise OR exercise therapies OR exercise rehabilitation OR exercises rehabilitation OR training resistance OR strength training OR exercise muscle stretching OR muscle stretching exercise* OR exercise movement technics OR pilates-based exercises OR stretching exercise  #4 injections intra articular OR intraarticular injections OR injections intraarticular OR injection intraarticular OR intraarticular drug administration OR intraarticular injection OR intra-articular injections OR injection intra-articular OR intra articular injections OR intra  #5 ((MH “Clinical Trials+”) OR “randomi?ed controlled trial*” OR clinical W3 trial OR double-blind OR single-blind OR triple-blind OR (MH “Placebo Effect”) OR (MH “Placebos”) OR (MH “Placebos”) OR placebo* OR random* OR (MH “Random Sample”) OR (MH “Study Design+”) OR latin square OR (MH “Comparative Studies”) OR (MH “Evaluation Research+”) OR (MH “Prospective Studies+”) OR follow-up stud* OR control* OR prospective* OR volunteer*) NOT (MH “Animal*”)articular injection  #1 AND #2 and #3 AND #4 AND #5 | **52** |
| **PEDro** | exercise* osteoarth* injection* knee LIMITS: CLINICAL TRIALS | **12** |
|  | **TOTAL** | **1047** |
