# Supplementary material for: Exercises plus intra-articular injection for knee osteoarthritis: a systematic review with meta-analysis
Source: Adv Rheumatol. 2025 Dec 10;66:5. doi: 10.1186/s42358-025-00458-3 (PMC13472110; doi:10.1186/s42358-025-00458-3)
Supplement: Supplementary file 2 [file 42358_2025_458_MOESM5_ESM.docx]

Supplementary Table 2 – Analysis of the quality of evidence (GRADE) for the variables that showed no benefit in the meta-analysis.

| **Outcomes** | **№ of participants (studies) Follow-up** | **Certainty of the evidence (GRADE)** | **Relative effect (95% CI)** | **Anticipated absolute effects** | |
| --- | --- | --- | --- | --- | --- |
| **IAI/HA + exercise versus IAI/HA (short term)** | | | | **Risk with Hialuronic acid (short term)** | **Risk difference with Hialuronic acid + exercise** |
| Function | 166 (3 RCTs) | ⨁◯◯◯Very low | - | - | SMD **0.67 higher** (0.58 lower to 1.93 higher) |
| **IAI/HA + exercise versus IAI/HA (medium term)** | | | | **Risk with Hialuronic acid (medium term)** | **Risk difference with Hialuronic acid + exercise** |
| Pain | 166 (3 RCTs) | ⨁◯◯◯Very low | - | - | SMD **0.14 lower** (0.8 lower to 0.52 higher) |
| Function | 166 (3 RCTs) | ⨁⨁◯◯Low | - | - | SMD **0.08 higher** (0.23 lower to 0.4 higher) |
| **IAI/HA + exercise versus IAI/HÁ (long term)** | | | | **Risk difference with Hialuronic acid + exercise** | |
| Function | 166 (3 RCTs) | ⨁◯◯◯Very low | - | SMD **0.53 lower** (1.22 lower to 0.15 higher) | |
| Pain | 166 (3 RCTs) | ⨁◯◯◯Very low | - | SMD **0.61 lower** (1.47 lower to 0.25 higher) | |
| **IAI with any medication + exercise versus IAI/SS + exercise (short term)** | | | | **Risk with Saline injection + exercise**  **(short term)** | **Risk difference with Intra-articular injection (any) + exercise** |
| Pain | 190 (4 RCTs) | ⨁◯◯◯Very low | - | - | SMD **0.11 higher** (0.52 lower to 0.74 higher) |
| Quality of life - physical function | 190 (4 RCTs) | ⨁⨁◯◯Low | - | - | SMD **0.27 lower** (0.57 lower to 0.02 higher) |
| Quality of life - mental health | 190 (4 RCTs) | ⨁⨁◯◯Low | - | - | SMD **0.33 lower** (0.68 lower to 0.02 higher) |
| **IAI with any medication + exercise versus IAI/SS versus exercise (medium term)** | | | | **Risk with Saline injection + exercise (medium term)** | **Risk difference with Intra-articular injection (any) + exercise** |
| Pain | 233 (5 RCTs) | ⨁◯◯◯Very low | - | - | SMD **0.54 higher** (0.09 lower to 1.17 higher) |
| Function | 233 (5 RCTs) | ⨁◯◯◯Very low | - | - | SMD **0.49 higher** (0.2 lower to 1.19 higher) |
| Quality of life - mental health | 233 (5 RCTs) | ⨁◯◯◯Very low | - | - | SMD **0.49 lower** (1.01 lower to 0.03 higher) |
| **IAI/PRP + exercise versus exercise (long term)** | | | | **Risk with Exercise (long term)** | **Risk difference with PRP + exercise** |
| Pain | 122 (2 RCTs) | ⨁◯◯◯Very low | - | The mean pain was **0** | MD **0.94 higher** (2.64 lower to 4.52 higher) |
| **IAI with any medication + exercise versus IAI/SS versus exercise (long term)** | | | | **Risk with Exercise (long term)** | **Risk difference with Intra-aticular injection (any) + exercise** |
| Pain | 268 (4 RCTs) | ⨁◯◯◯Very low | - | - | SMD **0.18 lower** (1.08 lower to 0.71 higher) |
| Function | 208 (3 RCTs) | ⨁◯◯◯Very low | - | - | SMD **0.78 lower** (1.92 lower to 0.36 higher) |
| Quality of life - mental health | 102 (2 RCTs) | ⨁⨁◯◯Low | - | The mean quality of life - mental health was **0** | MD **4.04 lower** (9.05 lower to 0.98 higher) |
| **IAI with any medication + exercise versus IAI with any medication without exercise versus IAI/SS versus exercise (medium term)** | | | | **Risk with Intra-articular injection**  **without exercise (medium term)** | **Risk difference with Intra-articular injection (any) + exercise** |
| Pain | 178 (4 RCTs) | ⨁⨁◯◯Low | - | - | SMD **0.05 higher** (0.25 lower to 0.35 higher) |
| Function | 178 (4 RCTs) | ⨁⨁◯◯Low | - | - | SMD **0.07 higher** (0.23 lower to 0.37 higher) |
| Range of Movement | 118 (2 RCTs) | ⨁◯◯◯Very low | - | The mean range of Movement was **0** | MD **3.92 lower** (8.63 lower to 0.78 higher) |
| **IAI/CE + exercise versus IAI/SS + exercise (short term)** | | | | **Risk with Saline + exercise (short term)** | **Risk difference with Corticosteroid + execise** |
| Pain | 130 (2 RCTs) | ⨁⨁◯◯Low | - | The mean pain was **0** | MD **3.94 lower** (17.56 lower to 9.69 higher) |
| Function | 130 (2 RCTs) | ⨁◯◯◯Very low | - | - | SMD **0.41 higher** (0.17 lower to 0.99 higher) |
| Quality of life - physical function | 130 (2 RCTs) | ⨁⨁◯◯Low | - | - | SMD **0.13 lower** (0.48 lower to 0.21 higher) |
| Quality of life - mental health | 130 (2 RCTs) | ⨁⨁◯◯Low | - | - | SMD **0.05 lower** (0.39 lower to 0.3 higher) |
| **IAI/CE + exercise versus IAI/SS + exercise (medium term)** | | | | **Risk with Saline + exercise (medium term)** | **Risk difference with Corticosteroid + execise** |
| Function | 130 (2 RCTs) | ⨁⨁◯◯Low | - | - | SMD **0.03 higher** (0.32 lower to 0.37 higher) |
| **IAI/ Botox + exercise versus IAI/HA + exercise (médium term)** | | | | **Risk with Hialuronic acid + exercise**  **(medium term)** | **Risk difference with Botulinum toxin + exercise** |
| Function | 96 (2 RCTs) | ⨁◯◯◯Very low | - | The mean function was **0** | MD **9.84 lower** (23.68 lower to 4 higher) |
| CI - confidence interval; MD - mean difference; IIA= injeção intra-articular; IIA/SS= injeção intra-articular com solução salina; IIA/CE= injeção intra-articular com corticoesteroide; IIA/AH= injeção intra-articular com ácido hialurônico; IIA/Botox= injeção intra-articular com toxina botulínica. | | | | | |
| **GRADE Working Group grades of evidence** **High certainty:** we are very confident that the true effect lies close to that of the estimate of the effect. **Moderate certainty:** we are moderately confident in the effect estimate: the true effect is likely to be close to the estimate of the effect, but there is a possibility that it is substantially different. **Low certainty:** our confidence in the effect estimate is limited: the true effect may be substantially different from the estimate of the effect. **Very low certainty:** we have very little confidence in the effect estimate: the true effect is likely to be substantially different from the estimate of effect. | | | | | |
